# Scanning for Therapeutic Targets within the Cytokine Network of Idiopathic Inflammatory Myopathies

**DOI:** 10.3390/ijms160818683

**Published:** 2015-08-11

**Authors:** Boel De Paepe, Jana Zschüntzsch

**Affiliations:** 1 Neuromuscular Reference Center, Laboratory for Neuropathology, 10K12E, Ghent University Hospital, 9000 Ghent, Belgium; 2 Department of Neurology, University Medical Centre, Göttingen University, 37075 Göttingen, Germany; E-Mail: j.zschuentzsch@med.uni-goettingen.de

**Keywords:** anakinra, avonex, cytokine, dermatomyositis, etanercept, inflammatory myopathy, infliximab, sifalimumab, tocilizumab, tumor necrosis factor α

## Abstract

The idiopathic inflammatory myopathies (IIM) constitute a heterogeneous group of chronic disorders that include dermatomyositis (DM), polymyositis (PM), sporadic inclusion body myositis (IBM) and necrotizing autoimmune myopathy (NAM). They represent distinct pathological entities that, most often, share predominant inflammation in muscle tissue. Many of the immunopathogenic processes behind the IIM remain poorly understood, but the crucial role of cytokines as essential regulators of the intramuscular build-up of inflammation is undisputed. This review describes the extensive cytokine network within IIM muscle, characterized by strong expression of Tumor Necrosis Factors (TNFα, LTβ, BAFF), Interferons (IFNα/β/γ), Interleukins (IL-1/6/12/15/18/23) and Chemokines (CXCL9/10/11/13, CCL2/3/4/8/19/21). Current therapeutic strategies and the exploration of potential disease modifying agents based on manipulation of the cytokine network are provided. Reported responses to anti-TNFα treatment in IIM are conflicting and new onset DM/PM has been described after administration of anti-TNFα agents to treat other diseases, pointing to the complex effects of TNFα neutralization. Treatment with anti-IFNα has been shown to suppress the IFN type 1 gene signature in DM/PM patients and improve muscle strength. Beneficial effects of anti-IL-1 and anti-IL-6 therapy have also been reported. Cytokine profiling in IIM aids the development of therapeutic strategies and provides approaches to subtype patients for treatment outcome prediction.

## 1. Introduction

The idiopathic inflammatory myopathies (IIM) are a heterogeneous group of autoimmune muscle disorders that include dermatomyositis (DM), polymyositis (PM), sporadic inclusion body myositis (IBM), and necrotizing autoimmune myopathy (NAM) [1]. The different subcategories of patients can be identified by examining the combination of clinical, histological and imaging features. DM can be present in children and adults, and is most often associated with subacute onset of proximal muscle weakness and characteristic skin manifestations. Skin involvement usually manifests with Gottron’s papules, the heliotrope eruption and mechanic’s hands. An increased risk of internal malignancies and interstitial lung disease (ILD) is associated with DM, and cardiac involvement with histologic evidence of inflammatory alterations is also described. PM presents more commonly in adults with subacute onset of symmetrical proximal muscle weakness. IBM is diagnosed most often in patients over 50 years of age. In most cases IBM is characterized by slowly progressive proximal and distal muscle weakness. A rare form of IIM is NAM, which presents as acute or subacute symmetrical proximal muscle weakness that develops after statin-use. As in DM, malignancies, ILD and cardiac involvement have also been reported. The different IIM subgroups present with distinct myopathological features. In DM, membrane attack complexes form on blood vessel endothelia, causing capillary loss and muscle ischaemia. In PM/IBM, nonnecrotic muscle fibers are invaded by autoaggressive CD8^+^ T-cells. The perforins and granzymes, they release, result in cytotoxic necrosis of the fibers [2]. In IBM, the inflammatory process is accompanied by degenerative phenomena and accumulation of abnormal protein aggregates inside the muscle fibers [3]. In NAM muscle tissue, inflammation is relatively rare and muscle fiber necrosis is the most prominent feature [4].

The chronic inflammation associated with the IIM is tightly regulated by cytokine expression. Cytokines are small proteins that, through their secretion, can shape immune cell function from a distance. The ultimate result is dependent not only upon cytokine concentration, but also on the local environment and the interactions with other cytokines and with non-immune cells. The sources of cytokines in human muscle tissues can be varied. Firstly, tissue-infiltrating immune cells are a rich source of cytokines, regulating each other’s activities and perpetuating the inflammatory response. Secondly, the muscle tissue constituents themselves can produce cytokines. It is well known that the interaction of muscle cells with immune cells can initiate and perpetuate immune responses in muscle tissue [5]. Muscle cells in culture constitutively express cytokines and can be stimulated to secrete an additional spectrum of pro-inflammatory cytokines, allowing them to participate to the immune reactions [6,7]. The blood vessel endothelium can also produce or present cytokines, allowing circulating responsive immune cells to find their way to tissue inflammation sites.

In this review, we describe the role of cytokines in the immune cell infiltration in IIM, and summarize common and emerging therapeutic approaches that display potential for the manipulation of cytokine networks in patients.

## 2. Roles of Cytokines in the Build-up of Inflammation in the IIM

In IIM; infiltrating macrophages; T-cells; B-cells; and dendritic cells (DC) are most often present in the affected skeletal muscles. These cells might contribute to a further inflammatory stimulus by perpetuated production of cytokines; and regulating the immune reactions in a profound way by recruiting; activating and shaping the activities of these tissue-infiltrating inflammatory cells.

### 2.1. Innate Immunity Activation

While autoimmunity is perceived to be mostly mediated by an adaptive immune response raised against self antigens, inappropriate activation of innate immune mechanisms has been shown to contribute substantially to the disease process. Innate or native immunity is the earliest line of host defense. In stressed cells undergoing necrosis, damage-associated molecular patterns (DAMPs or alarmins) that act as endogenous danger signals, are delivered to Toll-Like Receptors (TLR), leading to induction of nuclear factor κB (NF-κB) and its respective downstream pro-inflammatory cytokines including interferon (IFN) α/β, tumor necrosis factor (TNF) α, interleukin (IL)-1, IL-12 and IFNγ [8]. Innate immune reactions are mediated by phagocytic cells (neutrophils, macrophages, eosinophils), natural killer (NK) cells, the complement system, and the associated cytokines.

Many elements point to activated innate immunity in the IIM in general, and in DM in particular. TLR upregulation is strongly associated with the IIM. Recently in IBM, the “alarmin” High mobility group box-1 (HMGB1), a mediator of the innate immune response, has been demonstrated to play a crucial role in the inflammatory and degenerative network [9]. In DM, the attack on the vascular endothelium originates from complement-mediated humoral reactions and results in muscle and skin injury [10]. The type 1 IFN pathway is strongly associated with DM pathogenesis, and is upregulated in muscle, skin and in peripheral blood [11] and correlates with disease activity [12]. The cytokines IL-2 and IL-15, which share a receptor, have also been associated with innate immune reactions. IL-15 is involved in NK-cell development and proliferation, and is constitutively expressed in many cell types. In addition, IL-15 is an inducer of myocyte differentiation [13]. Barely expressed in healthy muscle, in DM/PM patients IL-15 is secreted by muscle cells through interaction with infiltrating T-cells [14]. In IIM tissue, IL-15 is expressed by infiltrating macrophages but not by T-cells [15]. Induction has been observed in both the muscle tissue and in serum of IIM patients, with IL-15 serum levels being significantly higher in adult DM than in PM [16]. IFNα and IL-2 were found to be 7.5-fold and five-fold increased in DM/PM sera respectively compared to healthy controls [17].

Innate and adaptive immunity are not separate entities, but are tightly connected through the activities of DC. DC are professional antigen-presenting cells divided into the more common myeloid DC and the IFNα-producing plasmacytoid DC. DC prime and activate naïve T-cells. Both myeloid and plasmacytoid, and immature and mature DC are present in IIM muscle [18] and in IBM, nonnecrotic muscle fibers are invaded by myeloid DC [19]. The cellular infiltrates in anti-HMG-CoA reductase-associated myopathy also contain DC [20].

### 2.2. Recruitment of Immune Cells

In healthy skeletal muscle, small numbers of resident immune cells are present that perform normal tissue immune surveillance activities. In chronic inflammatory muscle diseases however, mechanisms are activated that lead to massive extravasation of immune cells from the circulation to muscle tissue inflammatory sites. This is accomplished by the tagging of the local blood vessel endothelium with factors that retain responsive immune cells. Adhesion molecules and cytokines are upregulated, allowing the circulating mononuclear cells to attach and subsequently transmigrate through the blood vessel wall into the tissue. Matrix-metalloproteinases facilitate the migration of cells on their way to their targets [21].

Various chemotactic cytokines termed chemokines are specialized for recruiting mostly monocytes. An important class are the Monocyte Chemo-attractant Proteins (MCP): CCL2, CCL7, CCL8 and CCL13. Serum levels of CCL2 (two-fold) and CCL3 (around five-fold) are significantly higher in DM patients than in healthy controls, while CCL4 levels are no different [12]. In IBM, mRNA expression of CCL3 and CCL4 is significantly upregulated *versus* controls (123- and 125-fold) and protein upregulation is confirmed by immunohistochemistry [22]. The Macrophage inflammatory proteins (MIP): CCL3 and CCL4, are produced by macrophages, DC and T-cells. A microarray study showed CCL4 and CCL13 upregulation in PM muscle, but not in DM [23]. Other chemokines attract mostly T-cells. The IFNγ-induced chemokines CXCL9–11 provide highly selective lymphocyte recruitment attracting subsets of CD4^+^ and CD8^+^ T-cells [24]. In addition to other chemokines, CXCL9 has been demonstrated to be highly upregulated and localized to muscle fibers in IBM, and this increase could contribute to infiltration of immune cells [22,25]. An important attractant for DC in particular is CXCL12. Pre-DC express the corresponding receptor CXCR4 and become highly motile in response to CXCL12 [26]. Mature DC, that have been shown to respond also to CCL2 and CCL20 [27], accumulate in muscle tissue from IIM patients. Increased levels of CXCL12 are associated with the IIM, and the primary sources of the chemokine inside the muscle tissue are inflammatory cells and blood vessels. In DM muscle, perimysial B-cells express varying levels of CXCL12 [28]. In addition, CXCL12 is chemotactic for pre- and pro-B-cells, but does not attract mature B-cells [29]. An important and more universal attractant for B-cells is CXCL13 or B-cell-attracting chemokine 1 [30], which is especially prominent in the larger perimysial infiltrates and the follicular structures within DM muscle [31].

### 2.3. Activation of Macrophages

Functionally different macrophage lineages are present in IIM muscle: the classically activated pro-inflammatory M1 macrophages and the alternatively activated pro-tissue-repair M2 macrophages. The transformation of a circulating monocyte to classically activated M1 macrophages requires the exposure to two signals: priming by IFNγ followed by activation by TNFα directly or through an inducer of TNFα [32]. Both cytokines are expressed in IIM muscle. TNFα is a very prominent cytokine in IIM and high levels have been found both locally in the muscle as well as systemically in the serum of patients. TNFα levels were found eight-fold higher in DM/PM sera than in the sera of healthy controls [17]. In muscle lysates quantitative real-time PCR revealed that TNFα-mRNA was upregulated in PM (26-fold) and DM (12-fold) and in IBM (53-fold) compared to controls [22]. Both M1 and M2 macrophages are present in IIM muscle, and their relative proportions appear to be dynamic, altering with disease stage. M1 macrophages show increased inducible NO synthase activity, leading to an expanded capacity for producing cytotoxic NO. Tissue macrophages can organize into larger collections often surrounded by T-cells, forming an active and dynamic source of inflammatory cytokines that enhance each other’s activities.

### 2.4. T-Cell Differentiation and Activation

Clonal expansion of T-cells has been shown in PM/IBM muscle [33], indicating continuous antigen-driven immune reactions. Large numbers of CD4^+^ helper T-cells (Th) are present in IIM muscle. Differentiation into Th-subsets occurs through alternative activation of genes encoding transcription factors and cytokines, and suppression of genes associated with other lineages [34].

Many autoimmune diseases are dominated by Th1 immune-driven reactions, with prominent expression of the associated cytokines: IFNγ, IL-2, IL-12 and TNFα. IBM [35] as well as NAM [36] have been shown to display a prominent Th1 profile. IFNγ is three-fold increased in DM/PM patients, with even higher levels observed in IBM muscle [22]. IL-12 has been shown overexpressed in IBM [35], but others reported that IL-12 was not significantly altered in patients [17]. Expression of the chemokine receptor CXCR3 is associated with the Th1 lineage and DM/PM/IBM muscle contains high amounts of CXCR3^+^ CD4^+^ T-cells [37]. The receptor binds the IFNγ-inducible chemokines CXCL9, CXCL10 and CXCL11. CXCL9 has been shown to be upregulated in IBM [35] and PM, but not in DM samples [17]. CXCL10 serum levels have been found significantly increased in DM [12] and in DM/PM patients [17]. CXCL11 was found 5.5-fold (adult) and four-fold (juvenile) increased in DM sera, and protein levels correlated with disease activity [12]. Lower numbers of Th2 cells are found in IIM muscle. Cytokines mostly associated with the Th2 lineage are IL-4, IL-5, IL-6 and IL-13. IL-6 serum levels are significantly higher in DM than in control patients and correlate with disease activity [12].

IL-17 and the producing Th17 subset of CD4^+^ T-cells have been implicated in autoimmunity and are important pathogenic factors in rheumatoid arthritis and multiple sclerosis. Other Th17-associated cytokines are IL-21, IL-22, IL-23, IL-6 and TNF-related weak inducer of apoptosis (TWEAK). IL-17 activates monocytes and innate immunity, and is increased in DM and PM [38,39]. A study in 31 IIM patients showed that IL-17 serum levels inversely correlated with manual muscle test scores. No such correlation could be shown for the other cytokines tested, *i.e.*, IL-6, IL-10, IL-15, CCL2, CCL3 and CCL4 [16]. The Th17 pathway is important for maintaining chronic inflammation and is further regulated by cytokine expression. TWEAK stimulates IL-17 production by T-cells and favors Th17 differentiation. Increased TWEAK expression has been observed in IBM muscle, but not in PM/DM [40]. In the presence of IL-12 and TNFα, Th17 cells can convert into alternative Th1-cells that secrete both IFNγ and IL-17 [41]. Furthermore, TWEAK has been proposed to impair muscle differentiation, linking inflammation with myogenesis.

### 2.5. Maturation of B-Cells into Plasma Cells

Autoantibodies have been documented in DM, PM, IBM and NAM, and are increasingly recognized for their diagnostic potential. Antigen overexpression of known auto-antibodies has been noted in DM/PM [42]. Possibly, the enhanced tissue expression of these autoantigens, as part of the repair process in muscle, delivers them for recognition by autoantibodies, leading to ongoing autoimmune processes. Immunohistochemical staining showed the presence of fewer B-cells than differentiated plasma cells in IIM sections, with the plasma cells present in DM as well as in PM and IBM [43]. Interaction with T-cells in germinal centers of lymphoid structures have been shown to generate long-lived memory B-cells and effector plasma cells, and the lymphoid organizer LTβ is upregulated in CD20^+^ B-cells in DM tissues [44]. Clonal expansion suggests local and rapid transition of B-cells to antibody-producing plasma cells inside the muscle tissue. Local B-cell maturation in non-lymphoid tissues is a phenomenon observed also in other autoimmune diseases [45]. B-cell-activating factor (BAFF) is a crucial factor in the maintenance of B-cells, essential for the survival of mature B-cells. BAFF transcript has been reported to be markedly upregulated in DM (12-fold), PM (14-fold) and IBM (21-fold) compared to healthy samples [46]. BAFF expression by perimysial muscle fibers has been observed in DM [47]. Also, BAFF secreted by IFNα/β-stimulated DC activates B-cells and steers toward differentiation into (auto)antibody-producing plasma cells [48].

Different autoantibodies have been found associated with IIM subgroups. Anti-synthetase antibodies directed against cytoplasmic aminoacyl tRNA synthetases are the most prevalent. The anti-histidyl tRNA synthetase antibody termed Jo-1 is detected in 20% of DM/PM patients and is associated with a typical disease phenotype termed anti-synthetase syndrome [49]. With a lower frequency of ~5%, other antibodies are found, such as PL-7, PL-12, OJ, EJ, KS, Ha, and Zo, directed against different synthetases. Mi-2, directed against a component of the nucleosome remodeling deacetylase complex, is associated with a milder form of adult DM without interstitial lung disease or malignancy [50]. More severe IIM cases are associated with newly described auto-antibodies which mainly include enzymatic proteins *i.e.*, TIF1-γ, NXP-2, MDA5 and SAE [51]. In NAM, anti-3-hydroxy-3-methylglutaryl-coenzyme A reductase antibodies can be detected in statin-users [52]. Anti-signal recognition particle autoantibody (SRP) is associated with a rapidly progressive muscle weakness [53]. The muscle protein of 44 kDa (Mup44), which was identified as the cytosolic 5ʹ-nucleotidase 1A (cN1A) [54], represents a serological biomarker for systemic rheumatic disease and the IIM, including IBM [55]. These autoantibodies are referred to as myositis-specific and myositis-associated autoantibodies.

## 3. Conventional and Established Therapies for IIM

The therapeutic approach in myositis can be subdivided into initiating, maintenance and long-term therapy. For each treatment step, different immunosuppressive drugs are available. At the onset of disease, DM/PM/NAM are preferably treated with high-dose oral glucocorticosteroids (GC) or with intravenous methylprednisolone. In most patients GC are effective but the chance of relapses increases with tapering of the dose. Steroid-sparing drugs or second-line treatment such as azathioprine, methotrexate or intravenous immunoglobulins can be used for maintenance. Among the IIM, IBM is an exception as treatment has been shown mostly ineffective and remains controversial among experts.

### 3.1. General Treatment Recommendations for DM/PM/NAM

The accepted initial standard treatment for these IIM subtypes are GC. An aggressive approach with high doses is recommended in severe conditions in form of pulsed intravenous GC application, e.g., 250–500 mg prednisolone per day for 3–5 days. For milder clinical presentation and as a follow-up of the high-dose intravenous treatment, prednisone is given orally at 1 mg/kg bodyweight. The dosage is gradually reduced to a maintenance dose of 5–10 mg, to avoid disease relapses on the one hand, and adverse effects on the other hand. The most common adverse effects of GC treatment are edema, hypertension, potassium loss, insomnia, cataract/glaucoma and after long-term use osteoporosis, weight gain and steroid myopathy. The value of GC treatment in myositis has been described since 1950 [56] but, although accepted in several guidelines, has never been proven in rigorous randomized controlled trials [57]. Lately, the alternative application of monthly treatment with high doses of dexamethasone was compared to daily doses of prednisolone in a multicenter, double-blinded study. The dexamethasone application was not superior in effectiveness to daily prednisolone as first-line treatment of myositis but in total showed less frequent side effects [58]. As far as patients respond to GC therapy, a long-term maintenance non-steroidal immunotherapy can be started in severe cases. One Cochrane-Review analyzing the therapies for DM/PM is available [59]. The authors identified 14 relevant randomized-controlled trials (RCTs) from which four trials were excluded. The remaining 10 RCTs comprised 258 patients following heterogeneous treatment regimens, showed association with various side effects. Due to the lack of high quality RCTs, the selection of immunosuppressants is mainly empirical and the choice is based on the risk-to-benefit ratio, costs and the local guidelines.

In severe PM/DM/NAM, Azathioprine (AZA) can be given additionally to low dose prednisone. AZA is a purine antimetabolite that influences T- and B-cell proliferation. After testing the thiopurine methyltransferase (TPMT)-activity of the patient, the AZA therapy can be initiated with 50 mg/day and increased further under control of the blood count and liver enzymes to a target-dose of 2–3 mg/kg/day. For an effective treatment, the number of lymphocytes should be 600–1000/µL. The therapeutic effect is usually not expected before three to six months. The safety profile of AZA is well known for several autoimmune diseases. At the beginning (after 10–14 days), in ~15% of patients an idiosyncratic reaction occurs which then requires stopping AZA treatment; the hepatotoxicity and leukopenia are reversible when detected early and AZA is discontinued or reduced. In case of long-term treatment, the potential risk of developing malignancies increases. One should always be aware of the life-threatening interaction with allopurinol.

In case of AZA/prednisone failure or childbearing request, intravenous immunoglobulins (IVIG) can be tried as a second-line therapy. IVIG prepared from thousands of donors have numerous mechanisms of action including the interference with Fc receptors and Fc glycosylation, inhibition of cytokines and complement deposition or competition with autoantibodies [60]. The most convincing therapeutic effect was demonstrated for DM patients [61], but beneficial effects have also been shown in PM patients refractory to other treatments [62]. The initial dose is set to 2 g/kg, given either over five or two consecutive days. Due to its half-life of 18–32 days [63], maintenance dose of IVIG is infused regularly every four to eight weeks at a dose of 0.5–1 g/kg. In general, IVIG are well tolerated, but it is recommended that an IgA deficiency be ruled out before starting treatment. During the infusion, patients might complain of headache, chills, myalgia or chest complaints. Post infusion fatigue, fever or nausea may occur. Caution should be given to thromboembolic or hemolytic events, aseptic meningitis or to patients with renal abnormalities.

Methotrexate (MTX) is a folate inhibitor with a long immunosuppressive history for rheumatoid arthritis, and is therefore unfolding its effectiveness faster than AZA. MTX is a therapeutic option for severe PM/DM/NAM and in prednisolone refractory patients [64,65]. In addition, patients suffering from anti-synthetase syndrome with interstitial lung disease manifestations respond well to treatment with MTX [66]. Several doses and regimes have been described, but the most used application is an oral initial dose of 7.5 mg, although the subcutaneous application often shows more effectiveness and a better compatibility. After three weeks, MTX can be increased by 2.5 mg per week up to a total of 20–25 mg per week. MTX therapy should always be accompanied by an adequate folic acid supplementation. An important adverse effect is pneumonitis, sometimes difficult to distinguish from interstitial lung disease as seen in patients with Jo-1 positive anti-synthetase syndrome. Other side effects include hepatotoxicity, blood count alterations, gastrointestinal symptoms, renal abnormalities and malignancies.

Cyclosporin and mycophenolatmofetil (MMF) are alternatives for severe or prednisone-refractory PM/DM/NAM [67]. Cyclosporin disrupts the calcineurin pathway inhibiting T-cell proliferation. As a therapeutic reserve in infantile DM, doses of 2.5 to 5 mg/kg per day divided in two daily doses are given depending on plasma-levels and effectiveness. In adults, 2–4 mg/kg per day are sufficient to maintain the immunosuppressive effect after an initial dose of four to six mg/kg per day. Overall, cyclosporin-use is uncommon in IIM due to severe side effects such as hypertension, nephrotoxicity and central nervous system neurotoxicity. MMF is a prodrug of mycophenolic acid and suppresses B- and T-cell proliferation through a selective block of purine synthesis [68]. Meanwhile, some case reports describe the successful treatment of prednisolone refractory myositis with MMF [69,70] by using a dose of 1000–2000 mg twice daily. Complete blood counts should be performed weekly during the first month and afterwards once or twice monthly. MMF offers some pharmacological advantages compared to other immunosuppressants, e.g., the metabolism is independent from the TPMT-activity and hepatotoxicity is less common compared to AZA. The main adverse drug reactions (≥1% of patients) associated with MMF therapy include gastrointestinal symptoms, joint pain, infections, hyperglycemia, hypercholesterolemia, leukopenia, and anemia and, more rarely, pulmonary fibrosis or various malignancies. Cases of progressive multifocal leukoencephalopathy (PML) have been reported, but a causal relationship for PML remains under debate [71]. As for other immunosuppressants, females of reproductive potential must be made aware of possible congenital malformations.

In case of conventional treatment failure, cyclophosphamide is one of the strongest immunosuppressive drugs and has been used in DM/PM/NAM [72,73]. Cyclophosphamide is an alkylating agent of the nitrogen mustard group that must be processed in the liver to form the active aldophosphamide. Two ways of administration are available: 0.5–1.0 g/m^2^ can be given intravenously with an adequate co-medication, or 1–2 mg (cyclophosphamide)/kg/day orally. Its side effects include alopecia, bone marrow suppression, sterility, birth defects, mutations, and cancer. A strict monitoring of lymphocytes and neutrophils is necessary at day 7, 14 and 21.

Biological agents offer another option for treatment escalation in myositis. The monoclonal antibody Rituximab (RTX) mediates a depletion of B-cells by targeting CD20. The efficacy of RTX has been tested in a randomized, double-blind, placebo-phase trial in adult and pediatric myositis patients. An 83% success rate was achieved in a well-defined cohort of patients with treatment-refractory adult PM or adult or juvenile DM treated for 44 weeks [74,75]. Similar results were reported in a retrospective study of patients with severe, refractory PM or DM. The study demonstrated an objective improvement in the majority of patients with regard to creatine phosphokinase (CPK) and lung function tests [76]. These findings are supported by another retrospective study, which included 24 RTX-treated anti-synthetase syndrome patients with severe interstitial lung disease, reporting an improvement of pulmonary function test after a median of 52 months follow-up post-RTX [77].

### 3.2. General Treatment Recommendations for IBM

Although extensive research has been performed over the last years, debate is still ongoing as to whether IBM is primarily an inflammatory or a degenerative myopathy [78]. This has important implications, as a detailed understanding of disease pathomechanisms is crucial for the development of effective and specific drugs. A primary degenerative origin of IBM might explain the limited treatment efficacy of anti-inflammatory drugs. This assumption might be supported by a study that revealed that progression towards disability was exacerbated among patients receiving immunosuppressive drugs [79]. Although IBM was attributed with corticoid-resistance [80], individual patients have experienced a temporary clinical improvement under prednisolone therapy and no randomized studies to evaluate the steroid-efficacy have been conducted. Double-blinded studies that tested IVIG presented disappointing results. Authors concluded that treatment with IVIG may be either mildly effective on clinical outcome or even without any clinical relevance in IBM [81,82]. A significant effect of IVIG could however be achieved for the muscles of swallowing [62,83]. For MTX, a randomized, controlled study in 44 patients reported no improvement in muscle strength despite a significant decrease in creatine-kinase levels [84]. A combined therapeutical approach studied anti-T-lymphocyte globulin treatment followed by 12 months of oral MTX *versus* MTX alone in an open-label, randomized study. A slight improvement of distal upper extremity strength was noted for the combined therapy [85]. For MMF, a single case report with modest efficacy exists [86]. In a small study of 13 patients, alemtuzumab, a T-cell-depleting monoclonal antibody, was infused for four days with 0.3 mg/kg/d and showed to slow down the disease progression in some patients [87]. The promising results should be critically evaluated against possible severe side effects such as increased risk of opportunistic infection, autoimmune thyreoditis or idiopathic thrombocytopenic purpura. Taking the evidence together, no overall effective treatment is available today for IBM and according to the immunuosuppressive associated side-effects, most experts even abandon this option. At the moment, expectations rest upon an anti-myostatin approach that uses the humanized monoclonal antibody Bimagrumab [88]. This pilot trial, in which 11 patients received a single dose of bimagrumab and three received placebo, reported increased muscle mass after eight weeks and an improved six-minute walking distance after 16 weeks of dosing. Based upon these promising data, a large multicentre randomized, double blind, placebo-controlled study was initiated in 2013. The recruitment of 240 patients has been completed and the last infusion will be administered by the end of this year (the RESILIENT trial; NCT01925209). So far, the benefit remains unknown and the results on efficacy and safety are awaited by the myositis community.

## 4. Anti-Cytokine Agents and Their Potential for Treating IIM

Over the years, a more detailed understanding of the inflammatory process and the molecular pathways involved could provide alternative options for long-treatment resistant cases. A major approach for the development of targeted therapeutics for IIM has been attributed to cytokine-networks. The pro-inflammatory cytokines activated in the IIM offer attractive therapeutic targets. Cytokine-targeted therapies can be based upon (I) inhibition of cytokine production; (II) inhibition of cytokine action; (III) blockade of cytokine function; or (IV) receptor targeted therapy. These approaches necessitate the full evaluation of the repercussions of eliminating individual or several cytokines upon tissues. A thorough evaluation of the reduction/modification of cytokine patterns in inflammatory disease models, and the possible beneficial effects on health, could provide an excellent basis for designing therapeutic strategies for the future.

An inhibitor of cytokine gene transcription is thalidomide, which has multiple cellular and molecular effects [89] e.g., exhibiting anti-inflammatory and immunomodulatory properties [90]. One key mechanism is the destruction of TNFα mRNA [91]. In addition, the production of the proinflammatory cytokines IL-1, IL-6 and IL-12 from human mononuclear cells is blocked [92]. Originally, thalidomide was sold as a sedative and hypnotic drug but was withdrawn due to teratogenicity and neuropathy after several years [93]. Later, thalidomide had a revival as a drug against refractory multiple myeloma [94]. A few case reports describing the efficacy of thalidomide in patients with refractory IIM have been reported [95,96]. The teratogenic potential is aimed to be overcome by analogues of thalidomide such as lenalidomide with an even greater potency in inhibiting TNF production [97]. To date however clinical trials with thalidomide or lenalidomide have not been started.

One of the most sought-after approaches for reducing cytokine activity is to develop drugs that specifically bind and neutralize an individual cytokine, or the receptor it activates. This can be achieved by administering small-molecule antagonists of receptors, modified cytokines, or antibodies directed against cytokines or their receptors. Monoclonal antibodies are convenient therapeutic agents, not in the least by their excellent specificity, yet they come with important technical challenges. One of the most important is that they can elicit immunogenic responses in the patient. To circumvent this issue, engineered antibody constructs are continuously being developed. Starting from mouse antibodies, murine constant regions are replaced by human constant regions, creating chimeric antibodies. When all regions except the complementarity determining regions of the variable regions are of mouse-sequence origin, antibodies become humanized and ultimately fully human antibodies are created. Nonetheless, fully human sequence derived antibodies are, of course, less immunogenic, but can still induce immune responses. In rheumatoid arthritis, patients co-medicated with MTX and Golimumab, for instance, 16% were shown to have anti-drug antibodies [98].

Most important anti-cytokines agents, evidenced in patients with inflammatory disease and, when available, results from case reports and clinical trials in IIM (Table 1) [99,100,101,102,103,104,105,106,107,108,109,110,111,112,113,114,115,116,117,118] are listed hereunder.

**Table 1:** Results reported for anti-cytokine agents in refractory idiopathic inflammatory myopathies.

Compound and Treatment Regimen	Diagnosis/#Patients	Follow-up/Weeks	Clinical Outcome	Reference
**Anti-TNFα**				
infliximab 6 mg/kg 4-weekly or more frequent	jDM/5	32 to 130	I (5/5)	[99]
infliximab 10 mg/kg (week 0, 2, 6, 14)	DM/1	16	I (3/9); NC (4/9);W (2/9)	[100]
	PM/4			
	IBM/4			
infliximab 10 mg/kg (week 0, 2, 4)	DM/1	12	I (2/2)	[101]
	PM/1			
infliximab 10 mg/kg (week 20)	DM/1	66	I (2/2)	[102]
infliximab 10 mg/kg (week 14, 18, 22)	PM/1			
infliximab 10 mg/kg (week 0, 2, 6, 14, 22)	PM/2	26	I (2/2)	[103]
infliximab 10 mg/kg (week 0, 2, 4, 6, 9)	PM/1	69	I (1/1)	[104]
infliximab 5 mg/kg (week 0, 2, 6, 14, 18, 22)	PM/1	22	I (1/1)	[105]
infliximab 5 mg/kg weekly (0, 2, 6) every 8 weeks	DM + ILD/14		I (10/14); † (4/10)	[106]
infliximab 3 mg/kg (week 0, 2, 6,every 8) and or etanercept 25 mg twice weekly	DM/3	26	I (6/8)	[107]
	PM/5			
etanercept 50 mg weekly for 24 weeks	DM/8	24	I (6/11); NC (3/11); W (2/11)	[108]
	DM/3			
etanercept 25 mg twice weekly	PM/1	56	I (1/1)	[109]
etanercept 25 mg twice weekly	IBM/9	24 to 48	I	[110]
etanercept 25 mg twice weekly	DM/5	12	W (5/5)	[111]
etanercept 0.4 mg/kg twice weekly 1–12, 13–24 stop	jDM/6	12	I (3/6); NC (1/6); W (2/6)	[112]
**Anti-IFNα**				
sifalimumab 0.3–10 mg/kg every other week, 6 months	DM/26	14	I (38/51)	[113]
	PM/25			
avonex (βIFN1a) 30 µg weekly, 6 months	IBM/29	24	NC (27/29); W (2/29)	[114]
**Anti-IL-1**				
anakinra 100 mg daily	PM/6	48	I (7/15); NC (5/15); W (3/15)	[115]
	DM/4			
	IBM/5			
anakinra 100 mg daily	IBM/4	28	W (4/4)	[116]
anakinra 100 mg daily	PM/1 Jo-1positive antisynthetase syndrome	80	I (1/1)	[117]
**Anti-IL-6**				
tocilizumab 8 mg/kg every 4 weeks	PM/2	29 to 43	I (2/2)	[118]

Abbreviations: (juvenile) dermatomyositis ((j)DM), improved (I), sporadic inclusion body myositis (IBM), no change (NC), polymyositis (PM), DM/PM/IBM (DM/PM/IBM), worsened (W). Number of patients (**#**Patients), deceased (†).

### 4.1. Targeting TNFα

The important catabolic role of TNFα as a regulator of various chronic inflammatory diseases has made it a therapeutic target also in the IIM. Fortunately, murine studies showed that knocking out TNFα does not hamper skeletal muscle regeneration [119]. The cellular sources of TNFα in IIM are mostly the inflammatory cells, endomysial and perimysial mononuclear cells in DM/PM muscle samples express varying amounts of TNFα [120]. In DM, TNFα is also expressed by many endothelial cells and its soluble receptors TNF-R55 and TNF-R75 are increased in DM/PM serum compared to controls [121]. TNF-R75 is notably increased near inflammatory infiltrates in muscle from all IIM patient groups, and on the perimysial and perifascicular blood vessel endothelium in DM, even remote from inflammation [122].

In a strategy to inhibit posttranslational processing of TNFα, TNFα converting enzyme (TACE/ADAM17/CD156q) comes into the light. TACE is a multi-domain, transmembrane protein [123] that generates soluble forms of TNFα and other proteins from their membrane-bound precursors through a process that cleaves and releases the soluble ectodomain [124]. TACE inhibitors have been designed for treating rheumatoid arthritis, and have been tested in animal models and in clinical trials [125]. The initial promising TMI-005 (Apratastat) showed no hepatotoxicity, but the efficacy results prompted the company to terminate the trial [126]. As future treatment strategies, specific TACE inhibitors, which block formation of soluble TNF, are still awaited [127].

Many therapeutic anti-TNFα antibodies have been developed. Infliximab (Remicade), Adalimumab (Humira) and Golimumab (Simponi) are humanized monoclonal anti-TNF antibodies. Nerelimomab (Norasept) is a chimeric anti-TNFα monoclonal antibody. Certolizumab pegol is a PEGylated Fabʹ-fragment of a humanized antibody. Etanercept (Enbrel) is a fusion protein of TNFR2 bound to Fc-fragment of IgG_1_. Rheumatoid arthritis patients are being treated successfully with anti-TNFα agents for many years now [128]. In the IIM, several phase II clinical trials have been started up but, in general, studies suffer from low inclusion numbers and notably high drop-out rates mostly due to disease deterioration and adverse events (Table 1) [99,100,101,102,103,104,105,106,107,108,109,110,111,112]. However, it appears that anti-TNFα treatment could be of benefit to a subset of IIM patients. The identification of responsive patients remains difficult, as no specific marker has been identified yet that may predict the therapeutic outcome. Also, DM, PM and anti-synthetase syndrome have emerged in patients with other chronic inflammatory diseases for which they were taking anti-TNFα agents [129].

### 4.2. Targeting IFNα/β

An impressive IFN type 1-induced gene repertory is activated in DM, and milder upregulation of these genes is also observed in PM and IBM. Among the induced pathogenic gene products are major histocompatibility complexes, myxovirus resistance proteins, RNA helicases and cytokines [130]. Anti-IFN type 1 therapy would be able to calm down innate immunity-related reactions, neutralizing the IFN type 1-stimulated gene expression.

The immunomodulatory cytokine βINF counteracts the immunostimulatory effects of IFNγ and inhibits lymphocyte migration: this feature makes it a candidate therapeutic agent. The compound βINF1a (Avonex) has been tried in IBM and showed no significant effect on muscle strength [114]. Human anti-IFNα R monoclonal antibody MEDI-546 has been found safe in a Phase I trial with systemic sclerosis patients [131].

Several anti-IFNα antibodies have been developed. Sifalimumab, Rontalizumab and AGS-009 are monoclonal anti-IFNα antibodies. Results of a first Phase Ib clinical trial in IIM have been published. Six-months treatment with Sifalimumab suppressed the IFN type 1 gene signature in a cohort of DM and PM patients, including downregulation of IL-18 expression. Patient’s improvement of muscle strength correlated with greater neutralization of the type 1 IFN signature in blood and muscle [113]. In muscle biopsies from two selected treated patients, decreased T-cell infiltration was observed [114]. Blood analyses showed that five out of the 11 suppressed serum proteins were IFN-inducible, being sIL2R, CCL2, CCL8, BAFF and Ferritin, and that treatment reduced CXCL10 immunoreactivity in muscle from a PM patient. The medical community awaits results from other trials with great interest.

### 4.3. Targeting IFNγ

Unequivocal results have been published on IFNγ expression in the IIM [130], and several aspects of the specific role of IFNγ in the immunopathogenesis of the IIM remain elusive today. However, it seems its cross-talk with type 1 IFN could be an important aspect. In synergy with IFNα/β, IFNγ can shift immune responses toward Th1-mediated reactions, which, in turn, further perpetuate IFNγ production.

The humanized anti-IFNγ antibody Fontolizumab (HuZAF) has been developed, and has been shown efficacious for treating inflammatory bowel disease [132]. No results for treating IIM have been published.

### 4.4. Targeting BAFF

Of the TNF-family of cytokines other than TNFα, BAFF comes forward as an amenable target for treating the IIM [133]. Circulating BAFF levels have been found elevated in many patients with autoimmune diseases and, as a vital B-cell survival factor, it has emerged as a logical therapeutic target for combating B-cell hyperactivity. Targeting B-cells indirectly, rather than by a direct CD20-based approach (RTX) that has also been developed, has substantial advantages. Autoreactive B-cells tend to have a greater dependency on BAFF for their survival [134], which means that pathogenic B-cells could preferentially be eliminated this way. Non-selective B-cell depletion also risks harm to the protective capacities of the subset of regulatory B-cells [135]. In addition, BAFF influences T-cell function, more particular stimulating the Th1 [136] and Th17 [137] lineages. Targeting BAFF may thus counter both pathogenic B-cells and pathogenic T-cells in autoimmune diseases. BAFF has three receptors on B cells: B-cell maturation antigen (BCMA), TNFR homolog transmembrane activator and Ca^2+^ modulator and CAML interactor (TACI), and BAFF receptor (BR3). BCMA and TACI bind BAFF and the related cytokine a proliferation inducing ligand (APRIL). As APRIL contributes to the survival of plasma cells [138], blocking these receptors can neutralize both cytokines simultaneously, which may lead to an added neutralizing effect on auto-antibody production.

Several anti-BAFF agents have been developed. Fully human Belimumab (Benlysta, LymphoStat-B) and Tabalumab are monoclonal anti-BAFF antibodies. Blisbimod (AMG623) is a so-called peptibody *i.e.*, a fusion protein between IgG Fc and a BAFF-binding peptide sequence. Atacicept (TACI-Ig) is a receptor-Fc fusion protein that neutralizes both BAFF and APRIL signaling. Briobacept (BR3-Fc) is a fusion protein between BR3 and IgG Fc. Belimumab has been approved for treating systemic lupus erythematosus, where it has been shown to be safe and efficient for reducing peripheral blood B-cell numbers and circulating anti-dsDNA antibody levels [139]. Atacicept has also been shown to ameliorate systemic lupus erythematosus disease severity [140]. In a group of patients with acute rheumatoid arthritis, stable on MTX but with no adequate response to TNF inhibitors, Tabalumab showed indications of efficacy [141]. No trials have been started up with IIM patients so far.

### 4.5. Targeting IL-1

IL-1 is a crucial mediator of the inflammatory responses in chronic inflammatory conditions, and mediates muscle fiber damage in the IIM. In IIM muscle, IL-1 is mainly produced by activated macrophages, endothelial cells and muscle fibers [120,142,143]. IL-1α serum levels are increased in DM/PM patients, and correlates with disease activity [144,145]. The balance between IL-1 and its soluble receptor antagonist (IL-1Ra) is strictly regulated and imbalance between the two has been put forward as a disease aggravating factor [146]. IL-1 receptor antagonist (IL-1Ra) expression was shown to be increased in DM/PM sera [121], pointing to a regulatory function.

The IL-1β converting enzyme inhibitor Pralnacasan had already been tested in Phase IIb clinical trial on rheumatoid arthritis when results from an animal toxicology study showed liver abnormalities after a nine-month exposure at high doses. The trial was voluntarily discontinued.

Two receptor antagonists that block the effects of both IL-1α and IL-1β, have been developed. Anakinra (Kineret) is a recombinant soluble receptor IL-1Ra. Rilonacept is a dimer of IL-1R1 and IL-1RAcP linked to Fc-fragment of IgG_1_. The compounds Canakinumab and Gevokizumab are monoclonal anti-IL-1β antibodies. Anakinra has been tried in IIM and showed a response in some of the patients (Table 1) [115,116,117]. In a patient with familial Mediterranean fever and spondyloarthritis, subsequent myositis was successfully treated with Anakinra [147]. Gevokizumab is currently being tested in a randomized, double blind, placebo-controlled proof of concept study in patients with PM/DM/NAM (Integrated Research Approval System number 135286, European Union Drug Regulating Authorities Clinical Trials number 2012-005772-34).

### 4.6. Targeting IL-2

IL-2 expression, which is significantly increased in DM/PM sera [17], has been implicated in inflammatory diseases, activating T-cells as well as B-cells. A case report describes the development of myositis with endomysial inflammation in a patient treated with high dose IL-2 [148], illustrating the cytokine’s pro-inflammatory power. This patient received IL-2 for treating his advanced renal cell cancer, following the FDA approval that was issued due to the associated tumor regression and favorable outcome.

Chimeric Basiliximab and humanized Daclizumab are therapeutic monoclonal anti-IL-2Ralpha chain (CD25) antibodies. Daclizumab has been tested in several Phase II clinical trials for treating multiple sclerosis [149] and inflammatory bowel disease [150]. No results have been published in IIM patients.

### 4.7. Targeting IL-6

IL-6 is a cytokine with both pro- and anti-inflammatory properties. Classical IL-6 signaling mediates the activation of anti-inflammatory and regenerative pathways and is accomplished via the membrane-bound IL-6R. After binding IL-6, membrane-bound IL-6R recruits the signal-transducing receptor glycoprotein 130 kDa (gp130) [151]. Agonistic interaction with the soluble non-signal-transducing IL-6R on the other hand, can also occur. The IL-6/soluble IL-6R complex binds to a homodimer of gp130 on the cell surface, leading to so-called trans-signaling [152]. Trans-signaling activates a pro-inflammatory program. Immune cell recruitment is stimulated through induction of adhesion molecules and CCL2 expression by endothelial cells [153]. As most cell types, endothelial cells do not posses membrane-bound IL-6R, making them unresponsive to classical IL-6 signaling [154]. Healthy individuals have low levels of circulating IL-6 and higher levels of soluble IL-6R and gp130. Both IL-6 and soluble IL-6R are commonly upregulated in patients and autoimmune diseases, which are driven by IL-6 trans-signaling rather than classic signaling [155]. IL-6 serum levels are increased in DM and correlate with disease activity [12].

An impressive number of IL-6 targeting therapeutic antibodies have been developed. Tocilizumab, Sarilumab, Atlizumab (Actemra) and Sirukumab are therapeutic monoclonal anti-IL-6R antibodies. Olokizumab, Clazakizumab, Elsilimomab and Siltuximab are monoclonal anti-IL6 antibodies. BMS-945429 (ALD518) is an aglycosylated humanized monoclonal anti-IL-6 antibody. The Sgp130Fc agent is composed of the extracellular domain of human gp130 bound to the Fc-fragment of IgG_1_. While anti-IL-6 and IL-6R antibodies may not discriminate between signals initiated by membrane-bound or soluble receptors, Sgp130Fc selectively blocks IL-6 trans-signaling without affecting classic signaling [156].

One report describes successful Tocilizumab treatment in two patients with refractory PM (Table 1) [118]. Another report describes a patient with overlap syndrome that has been successfully treated recently [157]. In inflammatory bowel disease, Tocilizumab has also been shown to create clinical benefit [158]. In rheumatoid arthritis patients, administering Tocilizumab caused a shift in B-cell properties toward regulatory activities [159]. A Phase II trial with Tocilizumab for treating refractory DM and PM has been started (NCT02043548).

### 4.8. Targeting IL-12/IL-23

IL-12 is a heterodimeric cytokine composed of p35 and p40 subunits that stimulates Th1-cells. The related IL-23 is a heterodimeric Th17-associated cytokine that consist of p19 and p40 subunits, the latter it shares with IL-12. A role for IL-12 in myogenic differentiation has been reported [160], and the cytokine is overexpressed in IBM blood and muscle [35].

Ustekinumab and Briakinumab (ABT-874) are monoclonal anti-IL-12/IL-23 p40 subunit antibodies. Guselkumab and Tildrakizumab are therapeutic anti-IL-23 antibodies. Ustekinumab and Briakinumab are effective in psoriasis [161,162,163] and reduce both Th17 and Th1 cell numbers. Ustekinumab has also been tried in Crohn’s disease [164] and multiple sclerosis [165].

### 4.9. Targeting IL-17

The six IL-17 subforms A to F are pro-inflammatory cytokines, and their key source, *i.e.*, the Th17-cells, are abundant in inflammatory disease. IL-17 activates pro-inflammatory transcription factors, inducing cytokines, growth factors and other inflammatory mediators. Increased expression has also been shown in IIM muscle and serum [38].

Several therapeutic anti-IL17 antibodies have been developed, of which most are directed against the IL-17A form. Brodalumab, Secukinumab and AMG-827 are fully human monoclonal anti-IL17 antibodies. Ixekizumab, Perakizumab and LY2439821 are humanized monoclonal anti-IL17 antibodies. Secukinumab, Ixekizumab and Brodalumab have been evaluated in phase II clinical trials for rheumatoid arthritis [166], and phase III trials are ongoing. Their efficacy has been shown in psoriasis patients [167,168,169]. No results have been published in IIM patients.

### 4.10. Targeting CXCL10

The prominence of the CXCL10/CXCR3 axis in the IIM makes it an amenable target for therapeutic intervention. Our own results [37], since corroborated by others, have shown strong expression of CXCL10 and important infiltration by CXCR3^+^ cells in DM, PM as well as IBM. Inflammatory challenge has been shown to induce CXCL10 expression in myoblast cultures [170,171]. Vitamin D receptor agonists have been shown to counteract CXCL10 secretion by stimulated cells *in vitro* and their use as therapeutic agents in the IIM has been put forward [172].

Human monoclonal anti-CXCL10 antibodies have been developed and include MDX-1100 and BMS-936557. A Phase II double blind randomized study in patients with active ulcerative colitis showed improved clinical and histological parameters following eight weeks of BMS-936557 therapy [173]. A Phase II study of MDX-1100 and MTX combined for treating active rheumatoid arthritis has been completed and awaits publication of results (NTC01017367). To our knowledge, no trials for treating IIM have been started.

### 4.11. Targeting CCL2

CCL2 is prominently expressed in IIM, where it localizes to blood vessels and inflammatory cells. In PM and IBM, the macrophages and T-cells actively invading nonnecrotic muscle fibers express remarkably high levels of the chemokine [174]. The CCL2 expression profile points to an important role in myocytotoxicity directed against the muscle fibers in PM/IBM, as well as to a role in the endotheliopathy associated with DM, and makes it an amenable target for treating different IIM subgroups.

The development of anti-CCL2 therapeutics is currently at an early stage. A human monoclonal antibody against CCL2 termed Carlumab, and a humanized monoclonal antibody with high specificity to CCR2 termed MLN1202 have been generated. Carlumab is currently studied as an anti-cancer agent but has, to our knowledge, not yet been tested in inflammatory disease. A Phase IIa clinical trial with MLN1202 for treating rheumatoid arthritis showed no amelioration of synovial inflammation in active disease [175]. A proof of mechanism study will be started to assess if synovial inflammation can be reduced by the compound in atherosclerotic cardiovascular disease (NCT02388971).

## 5. Conclusions

There is a lack of consensus on standard care in IIM and no universal treatment recommendations have been put forward as yet. The use of GC remains the cornerstone of treatment, but the therapeutic armamentarium is in need of expansion, as no suitable therapy can be offered to large numbers of IIM patients. Also, therapy should be shaped to avoid recurrent infections and development of malignancies, as the approach most often requires lifelong treatment. The medical community awaits the reduction of risks associated with more specific immunosuppression. In this respect, cytokine-based treatment could offer the possibility of regulating the magnitude and duration of the immune response by restoring the regulatory immune balance.

Conventional immunosuppressive therapy reduces the expression of cytokines in responsive IIM patients. GC can act via the activated GC-receptor complex, which translocates as a dimer to the nucleus and activates the GC responsive element on GC-responsive genes with a subsequent increase of genes coding for anti-inflammatory proteins [176]. An additional mechanism is the suppression of the expression of responsive pro-inflammatory genes [177]. GC are able to strongly diminish the production of the “initial phase” cytokines IL-1β and TNFα and the “immunomodulatory” cytokines IL-2, IL-3, IL-4, IL-5, IL-10, IL-12 and IFNγ, as well as of IL-6, IL-8 and the growth factor GM-CSF [178]. Indiscriminate inhibition of the production of the “anti-inflammatory” cytokines, such as IL-10, might be responsible for treatment failures in myositis.

As they represent a relatively rare group of disorders, reports on the efficacy of anti-cytokine therapy in clinical trials enrolling IIM patients remain sparse. Interpretation of the results is not only difficult due to the small size of cohorts, but also due to the heterogeneity of the studied population. Beneficial effects have been reported for anti-TNFα, IFNα/β, IL-1 and IL-6 strategies in some of the patients, but it seems unlikely that in a heterogeneous and complex group of diseases, as is the case with IIM, targeting a single cytokine would lead to a meaningful amelioration of disease in every single case. Undoubtedly, the complexity of IIM subgroup-specific immunopathogenic mechanisms will necessitate further subtyping of patients, in order to predict therapeutic outcome, evolving to a more personalized therapeutic approach. It should also be taken into account that non-immune mechanisms can be equally crucial events in IIM pathology. In this respect, discussion on the primary degenerative or inflammatory origin of IBM is of interest. Also, in some of the patients, immunosuppression leads to complete removal of inflammation, yet without any clinical improvement. Also, the degree of inflammation does not always correlate with severity of muscle dysfunction. In IBM patients IVIG and/or prednisone treatment did not improve their muscle complaints. In these patients, muscle IL-1β, IFNγ, CXCL9, CCL3, CCL4 and TGFβ messenger levels were reduced, but TNFα and IL-6 levels were unaffected by the therapy [179]. From these results, one can conclude that reductions of individual cytokines do not necessarily predict therapeutic response, and that certain cytokines might be pathogenically more powerful and more difficult to neutralize.
